# Supplementary material for: Travelling spindles create necessary conditions for spike-timing-dependent plasticity in humans
Source: Nat Commun. 2021 Feb 15;12:1027. doi: 10.1038/s41467-021-21298-x (PMC7884835; doi:10.1038/s41467-021-21298-x)
Supplement: Supplementary file 1 — Supplementary Information [file 41467_2021_21298_MOESM1_ESM.pdf]

Supplementary Information

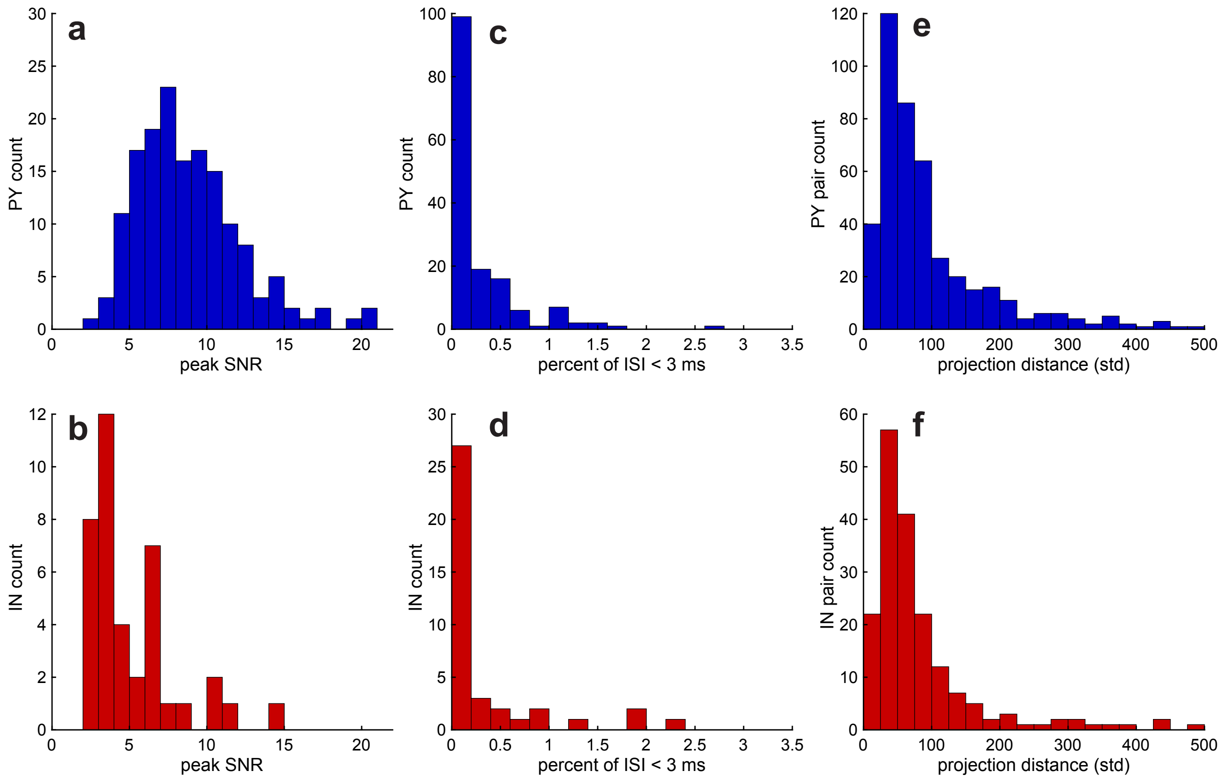

Supplementary Fig. 1. Unit quality and isolation metrics. a-b Histograms of peak signal-to-noise ratio of PY (**a**) and IN (**b**). **c-d** Histograms of percent of ISIs shorter than 3 ms for PY (**c**) and IN (**d**). Most cells had no, or very few, ISIs <3 ms. **e-f** Histograms of pairwise projection distances between all unit pairs detected on the same contact in units of standard deviation for PY (**e**) and IN (**f**). ISI=inter-spike interval.

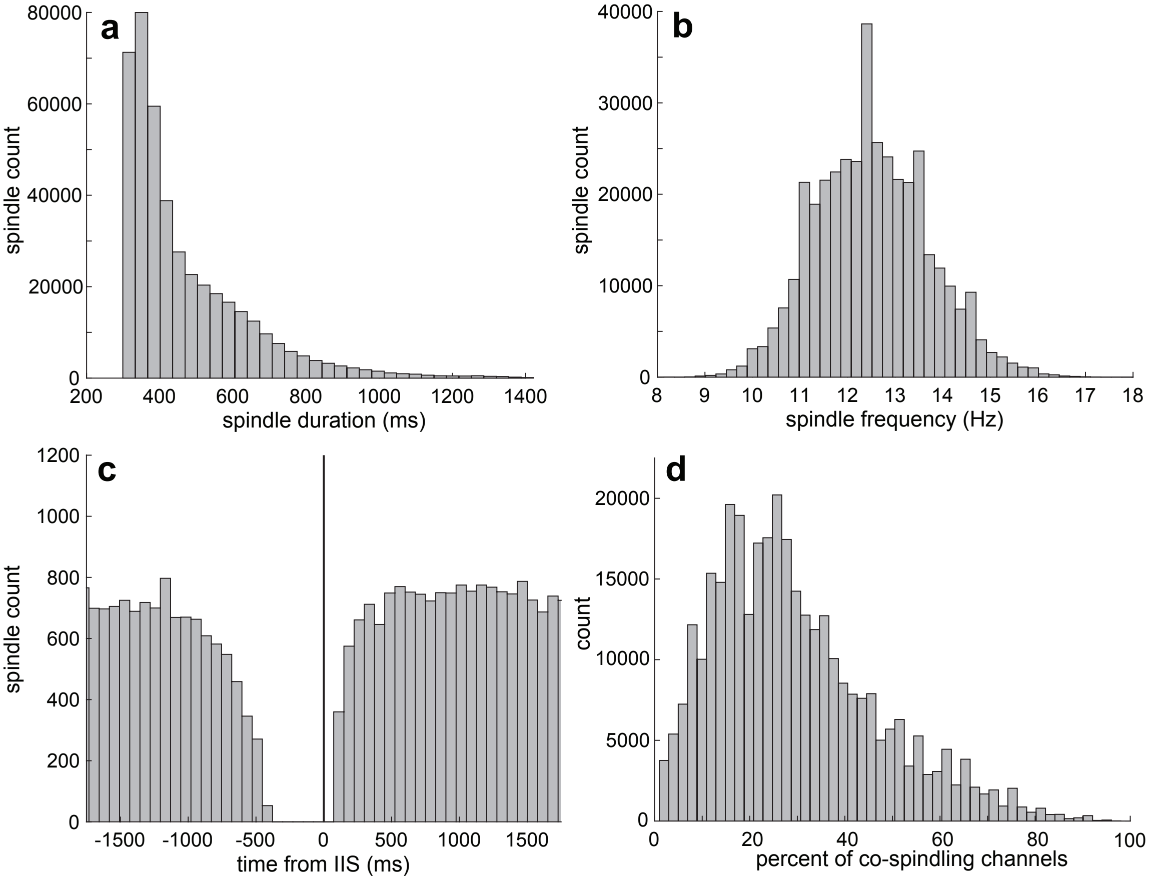

Supplementary Fig. 2. Spindle metrics. a Histogram of spindle durations. **b** Histogram of spindle oscillation frequencies. **c** Peri-IIS histogram of spindle onsets post-rejection of spindles that occurred within ±100 ms of IIS. **d** Percent of co-spindling channels when there was at least one spindle occurring. Note that about 15-40% of the channels were co-spindling in most events. IIS=inter-ictal spike.

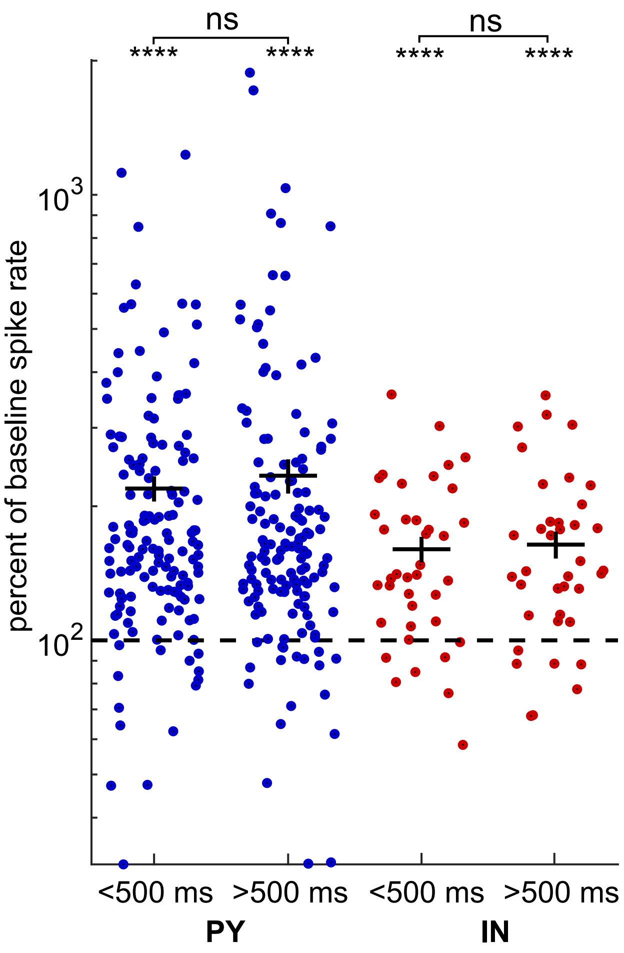

Supplementary Fig. 3. Unit spiking during shorter compared to longer spindles. Spike rates of PY (n=156) and IN (n=39) during concatenated spindle epochs as log-percent of baseline spike rate during shorter (<500 ms) spindles and longer (>500 ms) spindles. Baseline spike rate was computed as the spike rate during concatenated NREM epochs between spindles matched in number and duration to the spindles. Color circles show the mean firing rate of each unit. Solid horizontal lines show mean and vertical show SEM. Dashed horizontal line shows baseline spike rate (100%). The mean percent of baseline spike rate for PY during shorter spindles was 219.16±14.22% and longer spindles was 234.55±20.68%. These were both significant increases from baseline ($p_{PY,short}$=7e-22, $p_{PY,long}$=3e-21, Bonferroni-corrected α=0.025 for 2 spindle types, one sample two-sided Wilcoxon signed-rank test, $z_{PY,short}$=9.62, $z_{PY,long}$=9.45). The mean percent of baseline spike rate for IN during shorter spindles was 160.16±10.46% and longer spindles was 164.36±11.41%. These were also both significant increases from baseline ($p_{IN,short}$=4e-6, $p_{IN,long}$=2e-6, Bonferroni-corrected α=0.025 for 2 spindle types, one sample two-sided Wilcoxon signed-rank test, $z_{IN,short}$=4.61, $z_{IN,long}$=4.71). There was no significant difference between PY for shorter vs. longer spindles or IN for shorter vs. longer spindles (p_{PY}=0.40, p_{IN}=0.18, Bonferroni-corrected α=0.025 for 2 unit types, paired two-sided Wilcoxon signed-rank test, z_{PY}=0.84, z_{IN}=-1.34). ****p<0.0001.

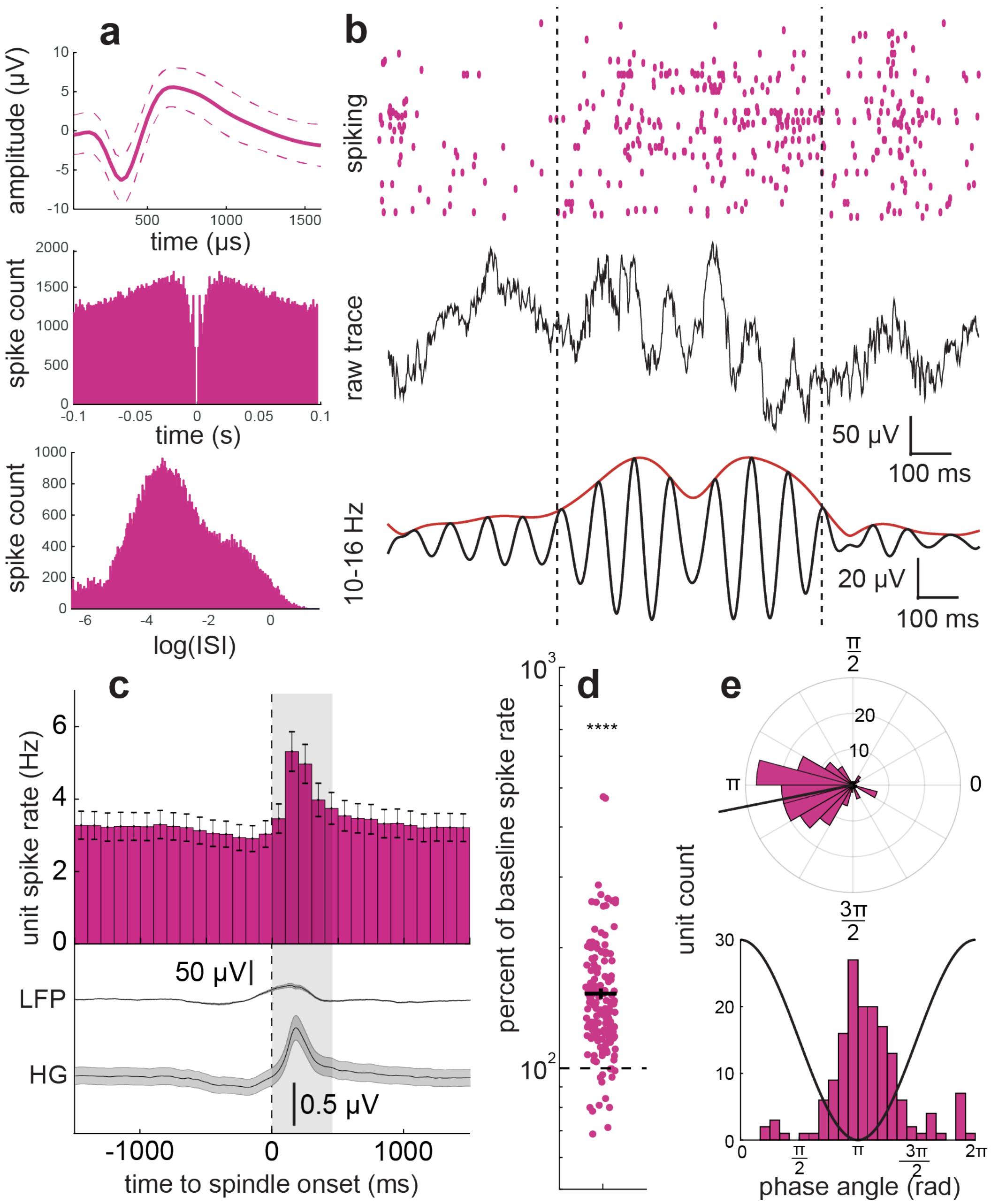

Supplementary Fig. 4. Multi-unit classification, spiking during spindles, and spindle phase preferences of spiking. a Average and standard deviation spike waveform (top), spike autocorrelation (middle), and ISI distribution (bottom) for an example MU. **b** Raw and 10-16 Hz bandpassed traces of an example spindle with raster plot of associated spiking of an example MU. **c** Mean MU spike rates, LFP, and HG envelope locked to spindle onsets at t=0 (n=158 units). Shaded gray box shows the mean spindle duration of 470 ms. Errors show SEM. **d** Mean MU spike rates during concatenated spindle epochs as log-percent of baseline spike rate (n=158 units). The mean and standard deviation baseline spike rate of MUs was 2.74±2.67 Hz. The mean and standard deviation spike rate of MUs during spindles was 3.70±3.38 Hz. MUs

had a significant increase of 153.69±4.54% (SEM) in the mean percent of baseline spike rate during spindles (p=1e-27, one sample two-sided Wilcoxon signed-rank test, z=10.90). Baseline spike rate of each unit was computed by concatenating randomly selecting NREM epochs in between spindles that were matched in number and duration to the spindles. Color circles show the mean firing rate of each unit. Solid horizontal lines show mean and vertical show SEM. Dashed horizontal line shows baseline spike rate (100%). **e** Polar and non-polar histograms show circular mean spindle phases of MU spikes (mean=3.33 rad). There was a significant spindle phase preference for 58.23% of MUs (α=0.05, Hodges-Ajne test with bootstrapping significance). One cycle of a spindle is superimposed on the non-polar histogram to visualize the phase-spike relationship. Black line extending from polar histogram shows circular mean. HG=high gamma, LFP=local field potential, MU=multi-unit. ****p<0.0001.

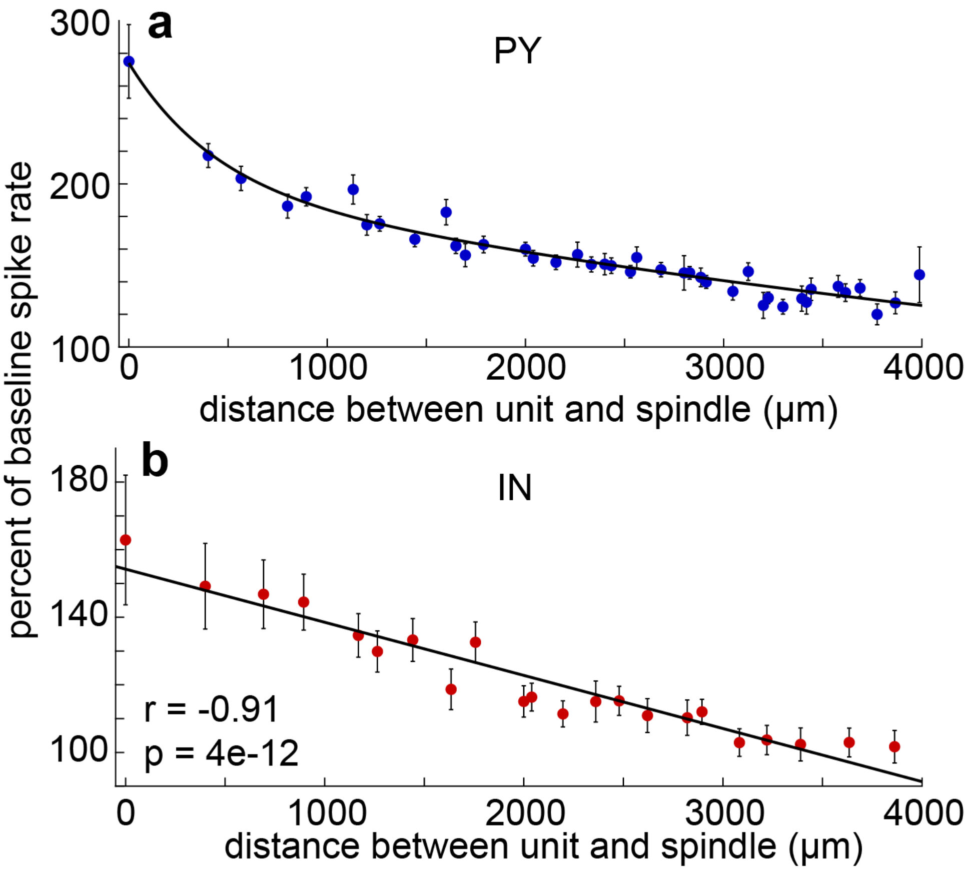

Supplementary Fig. 5. Unit spiking relative to distance to spindle. a Percent of baseline spike rate of PY for a co-located spindle (distance=0) or for spindles at progressively greater distances (n=50 unit-spindle channel pairs per point). Fit is two term exponential least squares regression (R^2=0.95). Note that PY unit spiking was greatest (~275% of baseline) when the unit channel was spindling (i.e., distance of 0) and decreased most sharply until ~1000 μm (~185% of baseline), at which point it decreased gradually (~125% of baseline at 4000 μm). **b** Same as **a** but for IN (n=50 unit-spindle channel pairs per point). IN unit spiking was also greatest when the unit's channel was spindling (~155% of baseline), and gradually decreased across space (~100% of baseline at 4000 μm) with a linear relationship (r=-0.91, p=4e-12, linear least squares regression, significance of the correlation coefficient). Therefore, the gradual decrease from ~1000-4000 μm for PY was similar to the gradual decrease from ~400-4000 μm for IN, and at shorter distances from the spindle PY spiking was greatly increased. Error bars show SEM.

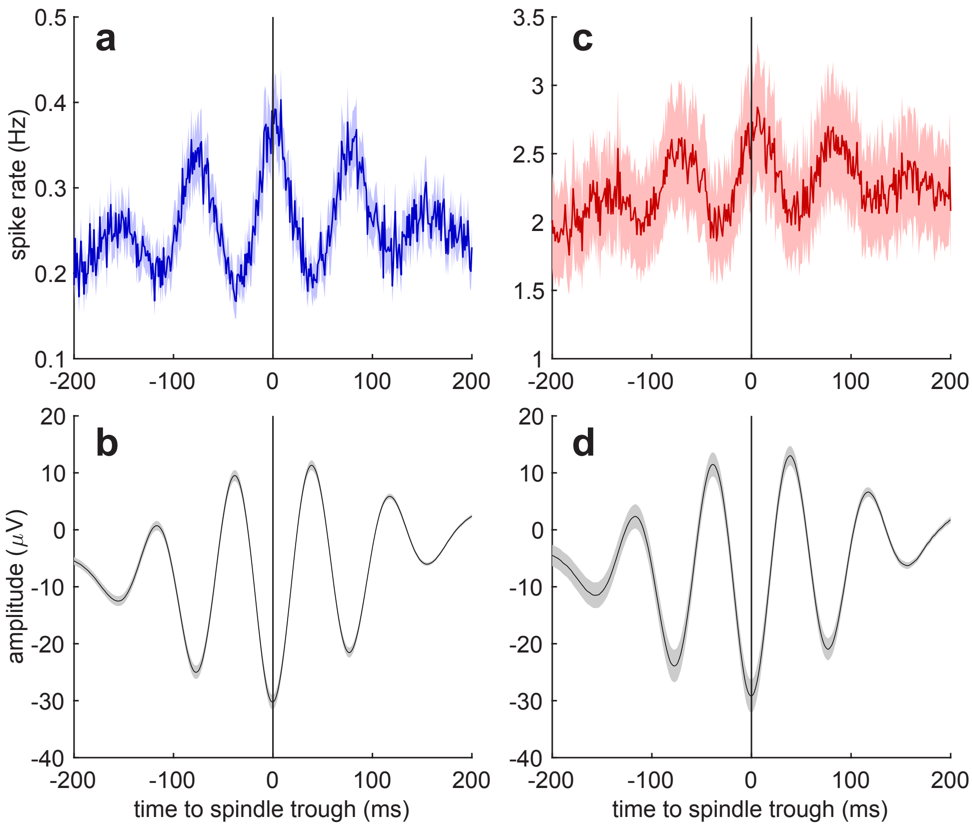

Supplementary Fig. 6. Spindle trough-locked unit spiking. a-b Mean spike rate of PY (**a**) and associated mean LFP (**b**) locked to all spindle troughs at t=0. **c-d** Same as **a-b** except for IN. Mean spike rates were Gaussian smoothed with a 50 ms window. Shaded error shows SEM.

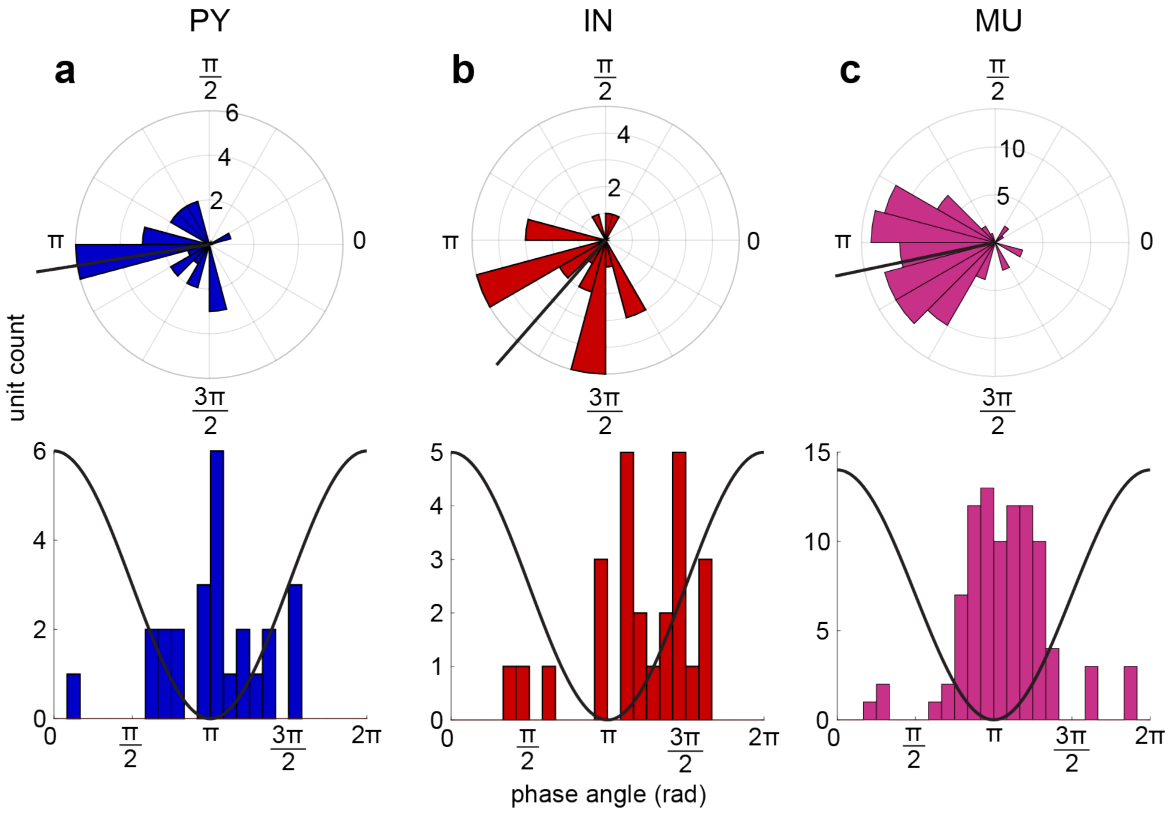

Supplementary Fig. 7. Spindle phase preference of units with statistically significant non-uniform spiking distributions. a-c Polar and non-polar histograms show circular mean spindle phases of spiking of PYs (**a**), INs (**b**), and MUs (**c**) with statistically significant non-uniform spike distributions (see **Methods**). Among the MUs with significant spindle phase preferences, the circular mean spindle phase of spiking was 3.34 rad. One cycle of a spindle is superimposed on non-polar histograms to visualize the phase-spike timing relationships. Black lines extending from polar histograms show circular means. MU=multi-unit.

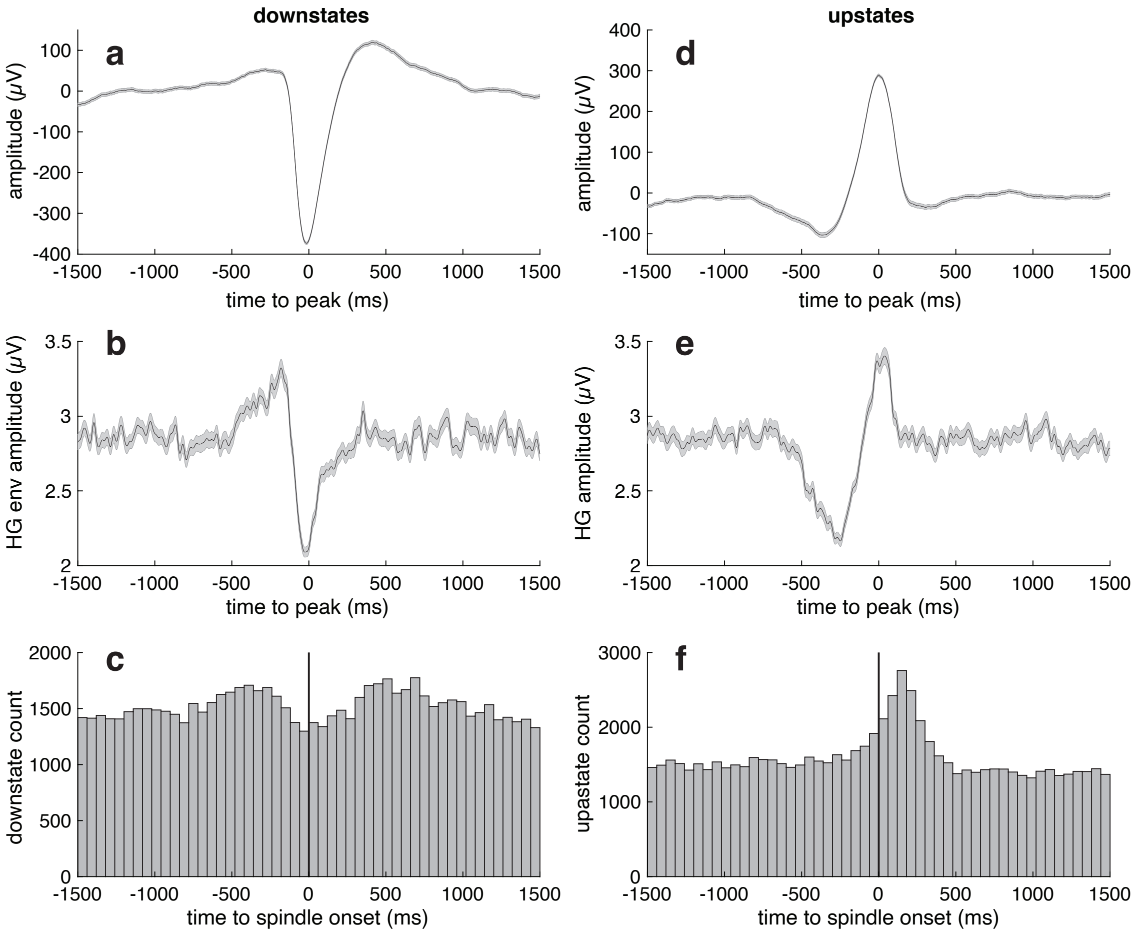

Supplementary Fig. 8. Downstates, upstates, and associated spindles. a-b Mean LFP (**a**) and HG analytic amplitude (**b**) of polarity corrected downstates for a representative channel. Means for both LFP and HG are relative to the LFP peak at t=0. Note that selected downstates were accompanied by upstates, and vice versa. **c** Peri-spindle onset time histogram of downstate peaks. **d-f** Same as **a-c** except for upstates. Shaded error shows SEM. HG=high gamma, LFP=local field potential.

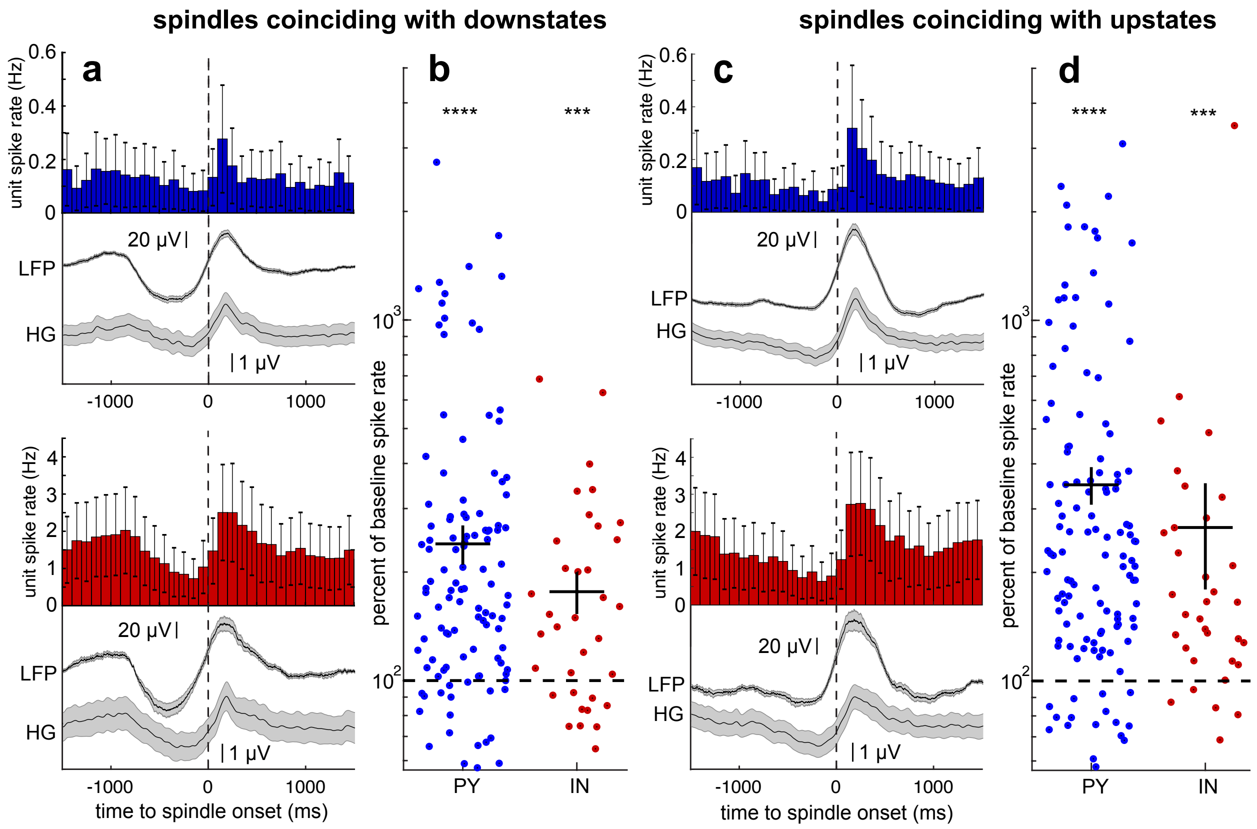

Supplementary Fig. 9. Unit spiking during spindles that occur with downstates or upstates. a PY (n=140) and IN (n=38) mean spike rates across units, and LFP and HG across unit channels relative to the onsets of spindles (t=0) that coincided with downstates, which were defined as spindles that had a downstate within 750 ms preceding their onsets. **b** PY (n=140) and IN (n=38) spike rates during concatenated spindles coinciding with downstates as a log-percent of baseline spike rates during concatenated NREM epochs matched in number and duration to the spindles. Solid horizontal lines show means. Dashed horizontal line shows baseline spike rate (100%). Spindles that coincided with downstates had a significantly increased spike rate from baseline (p_{PY}=7e-7, p_{IN}=0.001, Bonferroni-corrected α=0.025 for 2 unit types, one sample two-sided Wilcoxon signed-rank test, z_{PY}=4.97, z_{IN}=3.21). **c-d** Same as **a-b** except for spindles coinciding with upstates, which were defined as spindles that had an upstate within 500 ms following their onsets (n=125 PY, n=38 IN). Spindles that coincided with upstates also had a significantly increased spike rate from baseline (p_{PY}=2e-9, p_{IN}=0.003, Bonferroni-corrected α=0.025 for 2 unit types, one sample two-sided Wilcoxon signed-rank test, z_{PY}=6.02, z_{IN}=2.95). Only units that were detected on channels that had spindles that co-occurred with downstates (**a-b**) or upstates (**c-d**) were included. Note the similarities of the LFP traces and the similarities of the HG traces for spindles with downstates vs. upstates due to their systematic co-occurrence. These events were pooled for the analyses presented in the main text. HG=high gamma, LFP=local field potential. Errors show SEM. ****p<0.0001, ***p<0.001.

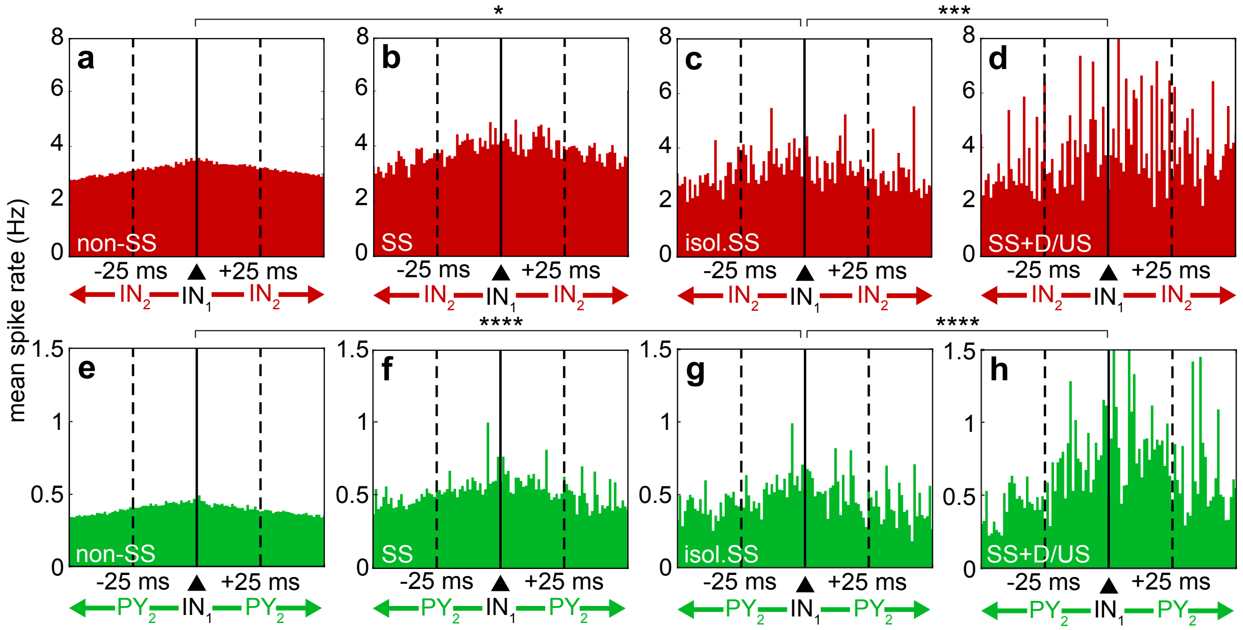

Supplementary Fig. 10. IN_1-IN_2 and IN_1-PY_2 co-firing during spindles. a In all pairs of IN_1-IN_2 recorded from different contacts, the firing of IN_2 is plotted relative to the time of IN_1 (at t=0) during non-spindle baseline epochs (**a**), spindles (**b**), isolated spindles that did not coincide with downstates or upstates (**c**), and spindles that coincided with down-to-upstates (**d**). **e-h** Same as **a-d** except for IN_1-PY_2. Solid vertical line shows t=0. Dashed vertical lines show the ±25 ms interval where paired pre- and post-synaptic spiking facilitates STDP. The mean and SEM co-firing rate within 25 ms for non-spindles vs. isolated spindles vs. spindles that coincided with down/upstates for IN_1-IN_2 was 3.31±0.17 vs. 3.60±0.27 vs. 3.97±0.35 Hz and for IN_1-PY_2 was 0.44±0.011 vs. 0.54±0.034 vs. 0.72±0.074 Hz. The co-firing rate during isolated spindles was significantly higher than non-spindles (p_{IN-IN}=0.016, p_{IN-PY}=3e-28, Bonferroni-corrected α=0.025 for 2 spindle types, paired two-sided Wilcoxon signed-rank test, z_{IN-IN}=2.41, z_{IN-PY}=11.01), and the co-firing rate during spindles that coincided with down/upstates was significantly higher than during isolated spindles (p_{IN-IN}=1e-4, p_{IN-PY}=2e-13, Bonferroni-corrected α=0.025 for 2 spindle types, paired two-sided Wilcoxon signed-rank test, z_{IN-IN}=3.80, z_{IN-PY}=7.37). *p<0.025, ***p<0.001, ****p<0.0001, paired two-sided Wilcoxon signed-rank test.

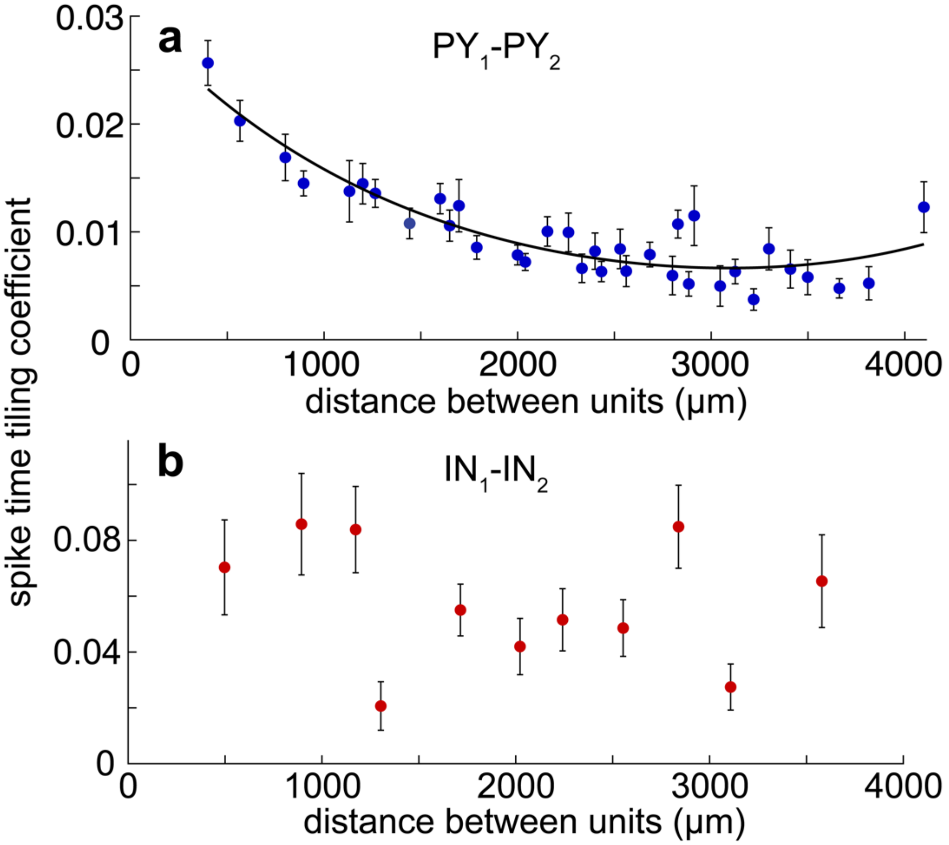

Supplementary Fig. 11. Unit pair co-firing across distance. The spike time tiling coefficient provides an alternative method for quantifying the tendency of units to fire in a correlated manner independent of firing rates (see **Methods**). We calculated the spike time tiling coefficient for PY_1-PY_2 (n=30 minimum unit pairs per point) and IN_1-IN_2 (n=30 minimum unit pairs per point) ±25 ms co-firing during all NREM periods included in this paper (consisting of stages N2 or N3). **a-b** Spike time tiling coefficient, which was used to measure the pairwise correlation within ±25 ms of spike trains for PY_1-PY_2 (**a**) and IN_1-IN_2 (**b**) as a function of the distance between units. PY_1-PY_2 correlations decreased with distance until ~2000 μm (R^2=0.83, two term exponential least squares regression). By contrast, IN_1-IN_2 correlations were larger overall and not dependent on distance at this scale. The PY_1-IN_2 and IN_1-PY_2 correlations are not reported since the spike time tiling coefficient test would not independently assess their correlations. Error bars show SEM.

Patient	a Spindle vs. baseline spike rate (%)		b Significantly phase-locked	
	PY	IN	PY	IN
1	166.50±10.28	162.34±16.26	16/69	15/23
2	342.64±49.48	260.65±91.75	3/46	4/8
3	127.73±10.98	145.20±31.23	4/16	6/7
4	232.37±24.05	189.93	2/14	0/1

Supplementary Table 1. Unit spiking and spindle phase-locking results for individual patients. a Mean spike rates during concatenated spindle epochs as a percent of spike rates during concatenated randomly selected non-spindle epochs matched in number and duration to the spindles. Error is SEM. No SEM was reported for patient 4 IN since there was only 1 IN detected in this patient. **b** Proportions of PY and IN with significant spindle phase preferences (α=0.05, Hodges-Ajne test with bootstrapping significance). IN=putative interneuron unit, PY=putative pyramidal unit, SEM=standard error of the mean.

Patient	a Paired co-firing: spindles vs. non-spindles				b Paired co-firing: spindles vs. shuff-spindles			
	PY_1-PY_2	IN_1-IN_2	PY_1-IN_2	IN_1-PY_2	PY_1-PY_2	IN_1-IN_2	PY_1-IN_2	IN_1-PY_2
1	1010/4638<br>21.78%	208/490<br>42.45%	410/1561<br>26.27%	547/1561<br>35.04%	295/4638<br>6.36%	140/490<br>28.57%	110/1561<br>7.05%	212/1561<br>13.58%
2	159/2910<br>5.46%	32/54<br>59.26%	111/430<br>25.81%	109/430<br>25.35%	151/2910<br>5.19%	36/54<br>66.67%	27/430<br>6.28%	8/430<br>1.86%
3	19/298<br>6.38%	19/36<br>52.78%	26/122<br>21.31%	32/122<br>26.23%	12/298<br>4.03%	12/36<br>33.33%	10/122<br>8.2%	6/122<br>4.92%
4	97/180<br>53.89%	N/A	12/14<br>85.71%	13/14<br>92.86%	38/180<br>21.11%	N/A	4/14<br>28.57%	9/14<br>64.29%
	c Paired co-firing: Both				d Ordered co-firing			
1	245/4638<br>5.28%	106/490<br>21.63%	89/1561<br>5.7%	193/1561<br>12.36%	14/77<br>18.18%	13/102<br>12.75%	9/64<br>14.06%	35/171<br>20.47%
2	145/2910<br>4.98%	28/54<br>51.85%	27/430<br>6.28%	8/430<br>1.86%	N/A	2/4<br>50%	N/A	N/A
3	8/298<br>2.68%	11/36<br>30.56%	5/122<br>4.1%	4/122<br>3.28%	N/A	3/10<br>30.00%	1/4<br>25.00%	1/3<br>33.33%
4	36/180<br>20%	N/A	4/14<br>28.57%	9/14<br>64.29%	4/17<br>23.53%	N/A	2/4<br>50.00%	3/9<br>33.33%

Supplementary Table 2. Unit pair co-firing results for individual patients. a-c Percent of unit pairs with significantly increased (α=0.001, bootstrapped significance) co-firing within 25 ms during spindles vs. non-spindles (**a**), spindles vs. shuff-spindles (**b**), and both (**c**). **d** Percent of unit pairs in **c**, which also had a minimum number of co-firing events of 10 within ±25 ms, with significant order preference of co-firing within ±25 ms (α=0.05, two-sided χ^2 test of proportions). N/A is indicated for Patient 4 IN_1-IN_2 since this patient had only 1 detected IN and otherwise when there was not a minimum of 10 co-firing events within ±25 ms.

Pair Type	Directionality	Spindles vs. non-spindles	Spindles vs. shuff-spindles	Both
PY-PY	sig one dir	18.04%	8.72%	6.25%
	sig both dir	6.83%	1.74%	1.45%
IN-IN	sig one dir	20.34%	18.62%	8.62%
	sig both dir	34.48%	23.10%	16.21%

Supplementary Table 3. Order preference of significant unit pair co-firing. For each PY-PY or IN-IN pair included in the co-firing analyses, also included was a pair of the same units but tested in the reversed order. Values are percent of sets of pairs that were significant in one direction only ("sig one dir") or both directions ("sig both dir") for spindles vs. non-spindles, spindles vs. shuff-spindles, and both tests.

	<10 ms, >0.90 coh	<10 ms, <0.50 coh	51-100 ms, >0.90 coh	baseline
PY_1-PY_2	0.96±0.85 Hz	0.78±0.86 Hz	0.60±0.68 Hz	0.25±0.06 Hz
IN_1-IN_2	4.25±3.44 Hz	3.76±3.01 Hz	2.56±2.51 Hz	2.68±0.48 Hz
PY_1-IN_2	4.04±3.91 Hz	3.03±3.75 Hz	2.41±3.04 Hz	1.40±0.15 Hz
IN_1-PY_2	1.05±1.16 Hz	0.53±0.86 Hz	0.64±0.68 Hz	0.28±0.02 Hz

Supplementary Table 4. Unit pair co-firing rates according to co-firing delay and spindle coherence. Mean and standard deviation unit pair co-firing rates during co-occurring spindles for shorter co-firing lags (<10 ms) and higher spindle coherence (>0.90), shorter lags and lower coherence (<0.50), longer lags (51-100 ms) and higher coherence, and longer lags and lower coherence during baseline NREM periods in between spindles.
