# Supplementary material for: Travelling spindles create necessary conditions for spike-timing-dependent plasticity in humans
Source: Nat Commun. 2021 Feb 15;12:1027. doi: 10.1038/s41467-021-21298-x (PMC7884835; doi:10.1038/s41467-021-21298-x)
Supplement: Supplementary file 3 — Descriptions of Additional Supplementary Files [file 41467_2021_21298_MOESM3_ESM.pdf]

Descriptions of Additional Supplementary Files

Supplementary Movie 1

Description: Travelling spindle with multiple propagation patterns on a sub-centimeter scale.
