# Supplementary material for: Travelling spindles create necessary conditions for spike-timing-dependent plasticity in humans
Source: Nat Commun. 2021 Feb 15;12:1027. doi: 10.1038/s41467-021-21298-x (PMC7884835; doi:10.1038/s41467-021-21298-x)
Supplement: Supplementary file 5 — Reporting Summary [file 41467_2021_21298_MOESM5_ESM.pdf]

nature research

Corresponding author(s): Prof. Eric Halgren
Charles W Dickey

Last updated by author(s): Dec 14, 2020

Reporting Summary

Nature Research wishes to improve the reproducibility of the work that we publish. This form provides structure for consistency and transparency in reporting. For further information on Nature Research policies, see our Editorial Policies and the Editorial Policy Checklist.

Please do not complete any field with "not applicable" or n/a. Refer to the help text for what text to use if an item is not relevant to your study.
For final submission: please carefully check your responses for accuracy; you will not be able to make changes later.

Statistics

For all statistical analyses, confirm that the following items are present in the figure legend, table legend, main text, or Methods section.

n/a	Confirmed	
☐	☒	The exact sample size (*n*) for each experimental group/condition, given as a discrete number and unit of measurement
☐	☒	A statement on whether measurements were taken from distinct samples or whether the same sample was measured repeatedly
☐	☒	The statistical test(s) used AND whether they are one- or two-sided<br>*Only common tests should be described solely by name; describe more complex techniques in the Methods section.*
☒	☐	A description of all covariates tested
☐	☒	A description of any assumptions or corrections, such as tests of normality and adjustment for multiple comparisons
☐	☒	A full description of the statistical parameters including central tendency (e.g. means) or other basic estimates (e.g. regression coefficient) AND variation (e.g. standard deviation) or associated estimates of uncertainty (e.g. confidence intervals)
☐	☒	For null hypothesis testing, the test statistic (e.g. F, t, r) with confidence intervals, effect sizes, degrees of freedom and P value noted<br>*Give P values as exact values whenever suitable.*
☒	☐	For Bayesian analysis, information on the choice of priors and Markov chain Monte Carlo settings
☒	☐	For hierarchical and complex designs, identification of the appropriate level for tests and full reporting of outcomes
☐	☒	Estimates of effect sizes (e.g. Cohen's d, Pearson's r), indicating how they were calculated

Our web collection on statistics for biologists contains articles on many of the points above.

Software and code

Policy information about availability of computer code

Data collection	Blackrock Microsystems NeuroPort System - used for data acquisition
Data analysis	Matlab R2019b - used to run all analyses<br>FieldTrip v2 - used to view local field potentials<br>NeuroPatt Toolbox v3.0 - used for the spatiotemporal analysis of spindle wave propagation patterns<br>Cyclic ColorMap v1.1.1 - used to produce circular phase color maps for spindle wave propagation examples<br>prop_test v1.0 - used when indicated to compute p-values for a chi-square test of proportions<br>Circular Statistics Toolbox v1.21 - used to compute circular means and circular-linear correlations<br>computeCohen_d v1.0 - used to calculate the Cohen's d<br>griddata (Matlab R2019b) - used for 2D biharmonic spline interpolation

For manuscripts utilizing custom algorithms or software that are central to the research but not yet described in published literature, software must be made available to editors and reviewers. We strongly encourage code deposition in a community repository (e.g. GitHub). See the Nature Research guidelines for submitting code & software for further information.

Data

Policy information about availability of data

All manuscripts must include a data availability statement. This statement should provide the following information, where applicable:

- Accession codes, unique identifiers, or web links for publicly available datasets
- A list of figures that have associated raw data
- A description of any restrictions on data availability

Figure source data are provided with this paper. The authors' data sharing agreement currently does not permit making the raw data publicly available. The raw data that support the findings of this study are available from the corresponding authors upon reasonable request.

Field-specific reporting

Please select the one below that is the best fit for your research. If you are not sure, read the appropriate sections before making your selection.

☒ Life sciences ☐ Behavioural & social sciences ☐ Ecological, evolutionary & environmental sciences

For a reference copy of the document with all sections, see nature.com/documents/nr-reporting-summary-flat.pdf

Life sciences study design

All studies must disclose on these points even when the disclosure is negative.

Sample size	All available archival data from patients who did not meet the exclusion criteria below were selected for analysis.
Data exclusions	All data exclusion criteria were pre-established. Patients were excluded when the recording array was implanted into epileptogenic tissue because the objective was to study the neurophysiology of healthy tissue, or when there was not at least 60 minutes of overnight NREM stage 2/3 sleep data, so that a sufficient amount of data from each patient was included. Channels were excluded when there were large amounts of noise or no units detected. Spikes, spindles, and baseline epochs were excluded when they were in close proximity to sharp, large amplitude transients in order to exclude artifacts and epileptiform activities.
Replication	Replication was across 4 patients, 156 putative pyramidal units and 39 putative interneuron units, 265 channels, and 340,743 spindles. All reported findings were successfully replicated.
Randomization	Participants were not separated into different experimental groups and epoch selection was performed the same way across patients. For each participant, control epochs were randomly selected from the same electrode and state.
Blinding	Participants were not separated into different experimental groups and so blinding was not relevant to the study. Control and experimental epochs were treated identically and in parallel by the analysis programs so there was no possibility of operator error.

Reporting for specific materials, systems and methods

We require information from authors about some types of materials, experimental systems and methods used in many studies. Here, indicate whether each material, system or method listed is relevant to your study. If you are not sure if a list item applies to your research, read the appropriate section before selecting a response.

Materials & experimental systems

n/a	Involved in the study	
☒	☐	Antibodies
☒	☐	Eukaryotic cell lines
☒	☐	Palaeontology and archaeology
☒	☐	Animals and other organisms
☐	☒	Human research participants
☒	☐	Clinical data
☒	☐	Dual use research of concern

Methods

n/a	Involved in the study	
☒	☐	ChIP-seq
☒	☐	Flow cytometry
☒	☐	MRI-based neuroimaging

Human research participants

Policy information about studies involving human research participants

Population characteristics	Participants were patients with long-standing medically-uncontrollable partial seizures who were candidates for surgical therapy (removal of the epileptogenic focus). They were undergoing invasive monitoring to localize seizure onset. The 4 participants ranged from 21-51 years old, 1 was female, and 3 were right handed.

Recruitment	Participants were invited to participate after they had been scheduled for intraoperative monitoring for completely clinical reasons. They were told that their participation or not had no relevance for their clinical treatment, would not provide any personal benefit, and would not change the duration or other parameters of their stay. Some patients may have declined to participate, but we do not know if this happened. If it did there is no reason to suspect that the organization of sleep was different in their brains compared to the ones who did agree.
Ethics oversight	Patients agreed to participate in these research studies after fully informed consent according to the Declaration of Helsinki guidelines as monitored by the Institutional Review Board at Partners HealthCare (Massachusetts General Hospital and Brigham and Women's Hospital).

Note that full information on the approval of the study protocol must also be provided in the manuscript.

his checklist template is licensed under a Creative Commons Attribution 4.0 International License, which permits use, sharing, adaptation, distribution and reproduction in any medium or format, as long as you give ppropriate credit to the original author(s) and the source, provide a link to the Creative Commons license, and indicate if changes were made. The images or other third party material in this article are included in e article's Creative Commons license, unless indicated otherwise in a credit line to the material. If material is not included in the article's Creative Commons license and your intended use is not permitted by atutory regulation or exceeds the permitted use, you will need to obtain permission directly from the copyright holder. To view a copy of this license, visit http://creativecommons.org/licenses/by/4.0/
